# Female Urinary Incontinence Evidence-Based Treatment Pathway: An Infographic for Shared Decision-Making

**DOI:** 10.1089/jwh.2021.0266

**Published:** 2022-03-11

**Authors:** Jessica L. McKinney, Laura E. Keyser, Samantha J. Pulliam, Tanaz R. Ferzandi

**Affiliations:** ^1^ School of Rehabilitation Sciences, Andrews University, Berrien Springs, Michigan, USA.; ^2^ Renovia, Inc., Boston, Massachusetts, USA.; ^3^ Tufts Medical Center, Boston, Massachusetts, USA.; ^4^ Urogynecology and Pelvic Reconstructive Surgery, Keck School of Medicine at University of Southern California, Los Angeles, California, USA.

**Keywords:** health education, female urinary incontinence, infographic, shared decision-making, care pathway

## Abstract

**Objectives:**

Urinary incontinence (UI) is a highly prevalent burdensome condition among adult females in the United States, yet rates of care-seeking, evaluation, and treatment are nonoptimal. Components of evaluation and treatment are informed by research and professional society guidelines; however, a visual representation of this guidance does not exist. The objectives of this study are to review the literature regarding female UI care and to synthesize this information into a graphical format to facilitate health education, health care delivery, and shared decision-making.

**Methods:**

We reviewed published society guidelines, position statements, and associated references from the American College of Obstetrics and Gynecology, the Women's Preventive Services Initiative, American Academy of Family Physicians, American College of Physicians, the Society of Urodynamics and Female Urology, the American Urological Association, and the American Urogynecologic Society, and searched PubMed for related literature. We synthesized these findings into an evidence-based infographic depicting female UI risk factors, influences on care-seeking and provision, screening, evaluation, and a stepwise treatment approach.

**Results:**

This study summarizes current evidence and professional guidelines related to female UI into a compelling visual format and accompanying narrative. The infographic is intended as a tool for patient education, clinical practice, and research to facilitate shared decision-making and health care delivery.

**Conclusions:**

Female UI is highly prevalent, yet diagnosis and treatment are suboptimal. Use of an evidence-based infographic may positively impact patient knowledge and certainty about UI treatment and support health care provider counseling and decision-making.

## Introduction

Urinary incontinence (UI) in women is a highly prevalent health condition. Approximately 50% of women age >50 years experience UI, and >20 million women in the United States have bothersome UI.^1,2^ These estimates are projected to rise, due in large part to the size of the aging demographic and the national obesity epidemic, both of which are associated with increased risk of UI.^3^ The scale of the problem, as well as the associated economic, psychosocial, and physical burdens of UI, establish this clearly as a public health issue necessitating a population-focused perspective.^4–6^

As few as 25% of women with UI in the United States seek care for their condition.^7^ Inversely, most women with UI in the United States do not discuss their symptoms with a health care provider (HCP), and thus do not initiate care. At an individual level, barriers to care-seeking include embarrassment, lack of knowledge of treatment options, feeling that the symptoms are not bad enough, and unwillingness to bring it up independently of being asked by their HCP.^8–10^ At a health systems level, structural barriers include time constraints, competing priorities, insufficient health workforce, and sociocultural barriers that limit patient accessibility to health services.^11–13^ In addition, economic barriers to care limit access and affordability, particularly for quality-of-life conditions, which may be deprioritized in the context of acute or life-threatening conditions.^14,15^ In recognition of the numbers of women with UI who do not seek or receive care, a 2018 systematic review explored the utility of proactive UI screening for women, and a subsequent Women's Preventive Services Initiative recommendation was issued, endorsing annual screening for UI for women, ages 18+, using validated survey tools.^16^

**FIG. 1.**
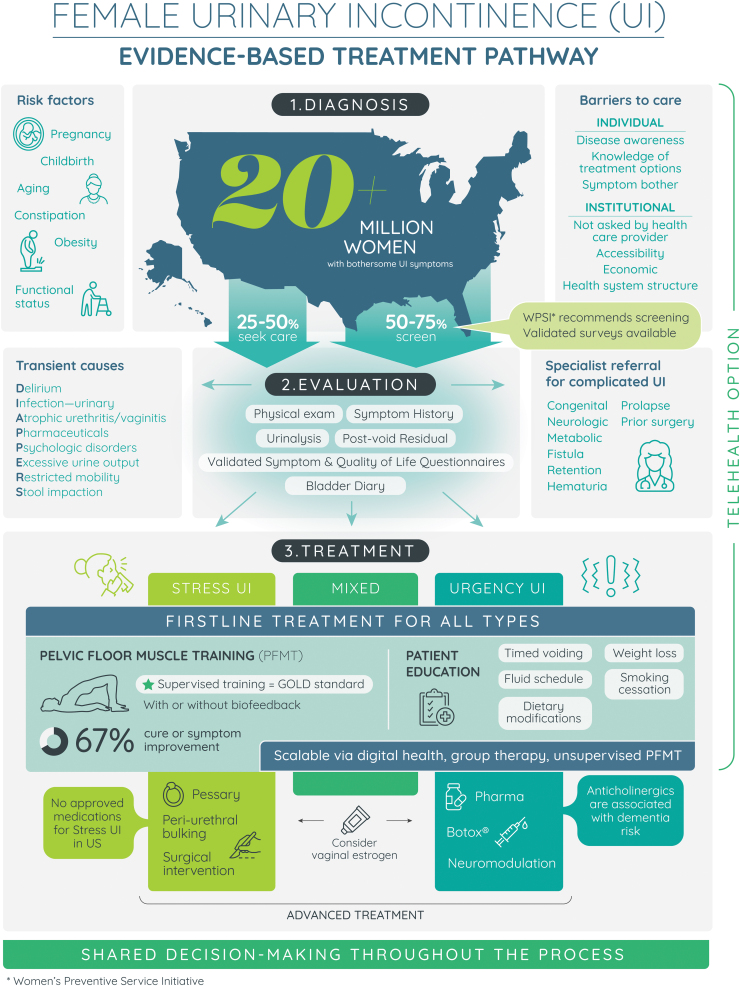
Infographic to support education and shared decision-making in screening, evaluation, and treatment for female urinary incontinence. ^©^Jessica L. McKinney et al. 2021; Published by Mary Ann Liebert, Inc.^42^

By integrating current evidence and synthesizing UI screening and treatment recommendations, this infographic was developed with aims (1) to improve patient and provider knowledge of this health condition and (2) to facilitate shared decision-making about treatment options, in line with professional guidelines (Fig. 1). Infographics convey important, often complex health information in a visually appealing display that may be quickly and easily understood.^17^ The American College of Obstetricians and Gynecologists (ACOG) endorses the use of decision aids, including infographics, citing evidence that these tools support patient-centered care and aid in counseling.^18,19^ The infographic presented in this study may be shared widely across professional organizations and health systems and utilized by general practitioners providing routine and/or well-woman care, as well as women's health or pelvic medicine specialists. A brief description and evidence to support the components of this infographic are summarized.

## Screening and Evaluation

**FIG. 2.**
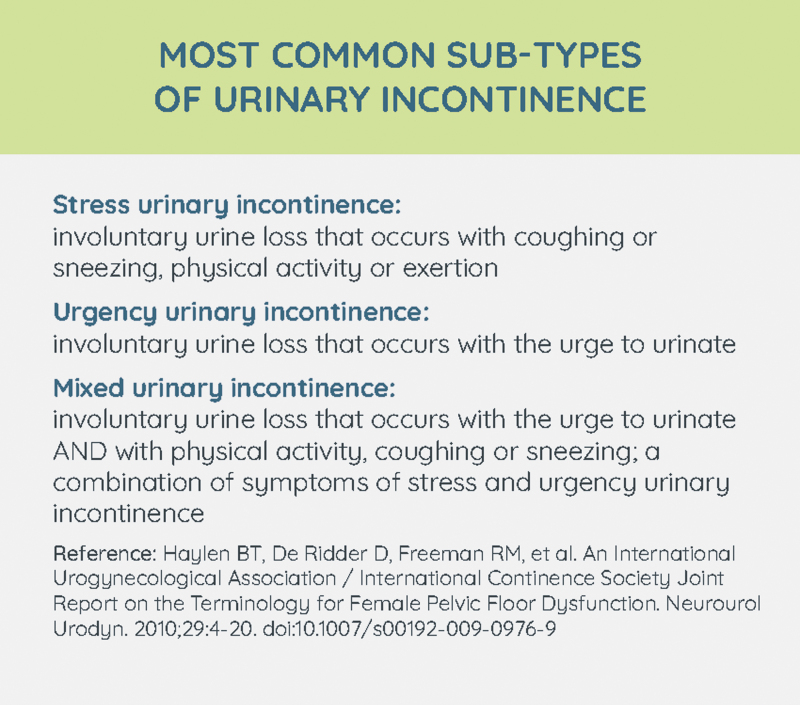
Definitions of stress, urgency, and mixed urinary incontinence.^43^

Whether through conventional “problem visit” care-seeking or implementation of standardized screening, women require preliminary evaluation of their UI. Screening may be facilitated using validated questionnaires, such as the 3-Incontinence Questions survey (3IQ), the Bladder Control Self-Assessment Questionnaire (B-SAQ), or the Michigan Incontinence Symptom Index (MISI).^16,20^ Evaluation entails a thorough subjective history, symptom evaluation, and risk factor assessment and may include the use of validated surveys. The Urogenital Distress Inventory-6 (UDI-6) and the International Consultation on Incontinence Questionnaire-Short Form for Urinary Incontinence (ICIQSF-UI) are common in research and clinical practice.^21,22^ Screening and symptom questionnaires may be used to determine presence of UI, suggest a provisional diagnosis of UI subtype or a more complicated cause, and/or to assess symptom severity and degree of bother.^20^ Physical examination, postvoid residual, and urinalysis are standard components.^23–27^ Potential causes of transient UI correspond to the acronym DIAPPERS (Delirium, Infection—urinary, Atrophic urethritis/vaginitis, Pharmaceuticals, Psychologic disorders, Excessive urine output, Restricted mobility, Stool impaction) and should be identified and addressed.^28^ Complicated UI due to congenital, neurological, or metabolic conditions, fistula, urinary retention, prolapse, or prior pelvic surgeries require specialist or subspecialist evaluation and care.^23,26^ Once these causes have been ruled out, it is important to differentiate among the major types of UI, including stress urinary incontinence (SUI), urgency urinary incontinence (UUI), or mixed urinary incontinence (MUI) (Fig. 2).^23–27^ Based on this diagnosis initial treatment recommendations can be made.

## Treatment: First-Line Care and Advanced Therapies

There is broad international and multidisciplinary agreement on most components of the UI care pathway, including adopting a stepwise approach.^26,29^ Universal consensus for first-line care for SUI, UUI, and MUI includes pelvic floor muscle training (PFMT) and other behavioral modifications such as bladder training, dietary changes, and/or fluid titration.^23–27^ PFMT is defined as “exercises for improving PFM strength, endurance, power and/or relaxation.”^30^ Level I evidence supports PFMT effectiveness and describes this intervention as most effective when performed under the supervision of a skilled HCP (supervised PFMT/sPFMT) for a period of at least 12 weeks.^31^ Recent publications highlight personal and structural barriers to implementing sPFMT for all women with UI.^8–13^ Gross limitations in the health workforce leave too few HCPs with the skills to provide sPFMT, compared with the numbers of women who require it.^11,12^ New care models are being tested and proposed to build capacity within the broader health system to care for women with UI. These include group-based PFMT, unsupervised PFMT, and the use of mobile technologies.^32–34^ Components of first-line care are considered minimal or no risk and may also play a role in multimodal therapy, implemented alongside advanced interventions.

Vaginal estrogen is recommended when vaginal atrophy is present with urinary symptoms.^23,25,26^ Beyond this and the first-line care described earlier, remaining treatment interventions for UI address either SUI or UUI (or the respective component of MUI). Although pessaries may be helpful for women with SUI, there are no FDA-approved medications for SUI available in the United States.^23,24,26^ Of those medications approved for UUI, anticholinergics are most often prescribed; however, HCPs must exercise caution when prescribing, in light of mounting evidence of increased dementia risk associated with chronic use.^35^ Beta-agonists are approved for urinary urgency and UUI, and are becoming more commonly prescribed due to fewer side effects, and thus greater tolerance and adherence among patients.^23,25–27^ Limitations in utilization of beta-agonists may be financial in nature, as they are more costly to patients and payers compared with anticholinergics.^36,37^ Advanced therapies for UI are often carried out at the specialist or subspecialist level although referral may be warranted at any time before this phase of care. For SUI, advanced therapies may include periurethral bulking agents and numerous surgical options.^23,24,26^ For UUI, these include intravesical Botox and in-office or implantable peripheral and sacral neuromodulation.^23,26,27^

Recent guidance indicates that remote care (*i.e.,* telemedicine) may be an appropriate vehicle for UI screening, initial evaluation, and the implementation of first-line care.^34,38–41^ In this context, physical examination, postvoid residual, and urinalysis may be deferred according to HCP interpretation of other evaluation components. The exception to the use of telemedicine to implement first-line care is the indication for additional in-person evaluation and/or referral. In all contexts—telemedicine or in-person care—evaluation and discussion of a patient's desire for treatment and their response to a given treatment should occur and be followed by shared decision-making about any next steps in care.^18^ An example of next steps is implementation of additional testing, such as urodynamics and/or cystoscopy, after first-line care that did not yield sufficient symptom improvement.

## Conclusion

This infographic synthesizes the literature and society recommendations in a visual format. Important factors preceding and concurrent with the patient–provider interaction are depicted, and the stepwise treatment pathway that may unfold over time is clearly illustrated. It may be useful for HCPs who want to engage in shared decision-making with their female patients, and readers are encouraged to print and share the infographic as a useful tool in patient education and clinical practice. Future study will examine use of this infographic in various settings to assess its impact on patient knowledge and certainty about UI treatment and HCP perceptions of its role in patient counseling and decision-making.

## Supplementary Material

Supplemental data
