# Supplementary material for: Female Urinary Incontinence Evidence-Based Treatment Pathway: An Infographic for Shared Decision-Making
Source: J Womens Health (Larchmt). 2022 Mar 11;31(3):341–6. doi: 10.1089/jwh.2021.0266 (PMC8972010; doi:10.1089/jwh.2021.0266)
Supplement: Supplemental data [file Suppl_FigS1.pdf]

FEMALE URINARY INCONTINENCE (UI)

EVIDENCE-BASED TREATMENT PATHWAY

Risk factors

- Pregnancy
- Childbirth
- Aging
- Constipation
- Obesity
- Functional status

1. DIAGNOSIS

20+ MILLION WOMEN with bothersome UI symptoms

- **25-50%** seek care
- **50-75%** screen

WPSI* recommends screening
Validated surveys available

Barriers to care

INDIVIDUAL

- Disease awareness
- Knowledge of treatment options
- Symptom bother

INSTITUTIONAL

- Not asked by health care provider
- Accessibility
- Economic
- Health system structure

Transient causes

- **D**elirium
- **I**nfection—urinary
- **A**trophic urethritis/vaginitis
- **P**harmaceuticals
- **P**sychologic disorders
- **E**xcessive urine output
- **R**estricted mobility
- **S**tool impaction

2. EVALUATION

- Physical exam
- Symptom History
- Urinalysis
- Post-void Residual
- Validated Symptom & Quality of Life Questionnaires
- Bladder Diary

Specialist referral for complicated UI

- Congenital
- Neurologic
- Metabolic
- Fistula
- Retention
- Hematuria
- Prolapse
- Prior surgery

TELEHEALTH OPTION

3. TREATMENT

STRESS UI | MIXED | URGENCY UI

FIRSTLINE TREATMENT FOR ALL TYPES

PELVIC FLOOR MUSCLE TRAINING (PFMT)

- ★ Supervised training = GOLD standard
- With or without biofeedback

67% cure or symptom improvement

PATIENT EDUCATION

- Timed voiding
- Weight loss
- Fluid schedule
- Smoking cessation
- Dietary modifications

Scalable via digital health, group therapy, unsupervised PFMT

ADVANCED TREATMENT

Stress UI:

No approved medications for Stress UI in US

- Pessary
- Peri-urethral bulking
- Surgical intervention

Mixed:

Consider vaginal estrogen

Urgency UI:

- Pharma
- Botox®
- Neuromodulation

Anticholinergics are associated with dementia risk

SHARED DECISION-MAKING THROUGHOUT THE PROCESS

* Women's Preventive Service Initiative

Infographic to support education and shared decision-making in screening, evaluation, and treatment for female urinary incontinence. ©Jessica L. McKinney et al. 2021; Published by Mary Ann Liebert, Inc.
McKinney JL, Keyser LE, Pulliam SJ, Ferzandi TR. Female Urinary Incontinence Evidence-Based Treatment Pathway: An Infographic for Shared Decision-Making. J Womens Health (Larchmt). 2021 Nov 5. doi: 10.1089/jwh.2021.0266. Epub ahead of print. PMID: 34747662.
